# Network analysis of trauma in patients with early-stage psychosis

**DOI:** 10.1038/s41598-021-01574-y

**Published:** 2021-11-23

**Authors:** Young-Chul Chung, Je-Yeon Yun, Thong Ba Nguyen, Fatima Zahra Rami, Yan Hong Piao, Ling Li, Bomi Lee, Woo-Sung Kim, Jing Sui, Sung-Wan Kim, Bong Ju Lee, Jung Jin Kim, Je-Chun Yu, Kyu Young Lee, Seung-Hee Won, Seung-Hwan Lee, Seung-Hyun Kim, Shi Hyun Kang, Eui Tae Kim

**Affiliations:** 1 Department of Psychiatry, Jeonbuk National University Medical School, 20, Geonji-ro, Jeonju, 54907 Republic of Korea; 2 Research Institute of Clinical Medicine of Jeonbuk National University-Biomedical Research Institute of Jeonbuk National University Hospital, Jeonju, 54907 Republic of Korea; 3 Department of Psychiatry, Jeonbuk National University Hospital, Jeonju, 54907 Republic of Korea; 4 Seoul National University Hospital, Seoul, 03080 Republic of Korea; 5 Yeongeon Student Support Center, Seoul National University College of Medicine, Seoul, 03080 Republic of Korea; 6 Brainnetome Center and National Laboratory of Pattern Recognition, Institute of Automation, Chinese Academy of Sciences, Beijing, 100190 China; 7 CAS Center for Excellence in Brain Science and Intelligence Technology, University of Chinese Academy of Sciences, Beijing, 100049 China; 8 Department of Psychiatry, Chonnam National University Medical School, Gwangju, 61469 Republic of Korea; 9 Department of Psychiatry, Inje University Haeundae Paik Hospital, Inje University College of Medicine, Busan, 48108 Republic of Korea; 10 Department of Psychiatry, The Catholic University of Korea, Seoul St. Mary’s Hospital, Seoul, 07345 Republic of Korea; 11 Department of Psychiatry, Eulji University School of Medicine, Eulji University Hospital, Daejeon, 35233 Republic of Korea; 12 Department of Psychiatry, Eulji University School of Medicine, Eulji General Hospital, Seoul, 01830 Republic of Korea; 13 Department of Psychiatry, Kyungpook National University School of Medicine, Daegu, 41944 Republic of Korea; 14 Department of Psychiatry, Inje University College of Medicine, Goyang, 10380 Republic of Korea; 15 Department of Psychiatry, Korea University College of Medicine, Guro Hospital, Seoul, 08308 Republic of Korea; 16 Department of Psychosocial Rehabilitation, National Center for Mental Health, Seoul, 04933 Republic of Korea; 17 Department of Psychiatry, Seoul National University Bundang Hospital, Seongnam, 13620 Republic of Korea

**Keywords:** Medical research, Network topology

## Abstract

Childhood trauma (ChT) is a risk factor for psychosis. Negative lifestyle factors such as rumination, negative schemas, and poor diet and exercise are common in psychosis. The present study aimed to perform a network analysis of interactions between ChT and negative lifestyle in patients and controls. We used data of patients with early-stage psychosis (n = 500) and healthy controls (n = 202). Networks were constructed using 12 nodes from five scales: the Brief Core Schema Scale (BCSS), Brooding Scale (BS), Dietary Habits Questionnaire, Physical Activity Rating, and Early Trauma Inventory Self Report-Short Form (ETI). Graph metrics were calculated. The nodes with the highest predictability and expected influence in both patients and controls were cognitive and emotional components of the BS and emotional abuse of the ETI. The emotional abuse was a mediator in the shortest pathway connecting the ETI and negative lifestyle for both groups. The negative others and negative self of the BCSS mediated emotional abuse to other BCSS or BS for patients and controls, respectively. Our findings suggest that rumination and emotional abuse were central symptoms in both groups and that negative others and negative self played important mediating roles for patients and controls, respectively.

*Trial Registration*: ClinicalTrials.gov identifier: CUH201411002.

## Introduction

Network analysis has been employed to investigate a) psychotic experiences^1^, potential pathways between psychotic symptoms and environmental risk factors or childhood trauma (ChT)^2^, and transdiagnostic experiences surrounding auditory verbal hallucinations^3^ in b) general population samples as well as interactions among a wide array of psychotic symptoms^4^ or among positive, negative, and depressive symptoms^5^, prediction of treatment responses^6^, negative symptom systems^7^, and pathways linking psychotic symptoms with ChT^8^ or post-traumatic stress^9^ in psychosis or schizophrenia. To date, only one study on the issue of trauma in psychosis^8^ has used a network approach; that study was based on data from patients but not from healthy controls.

Rumination is a repetitive and negatively valenced thinking style characterized by “the tendency to repetitively analyze one’s problems, concerns, and feelings of distress without taking action to make positive changes”^10^. Several studies have explored the relationships of rumination with depression^11^, negative symptoms^12^, positive symptoms^13^, and suicidality^14^ in psychosis. People with psychosis reported extreme negative evaluations of both self and others^15^. Furthermore, individuals with psychosis showing suicidal ideation held more negative evaluations of self and others than did those without suicidal ideation^16^. Moreover, in individuals with psychosis, negative beliefs following trauma were closely associated with psychotic experiences^17^. Patients with schizophrenia tend to have poor diets, characterized by high intake of saturated fat and low consumption of fiber and fruit^18^, and to have lower levels of physical activity compared with the general population^19^. One study reported that physical abuse was linked to elevated systolic blood pressure, whereas emotional abuse and neglect in women were linked to overweight in patients with schizophrenia^20^. Also in a non-clinical population, individuals with adverse childhood experiences were at increased risk of poor health outcomes such as physical inactivity, overweight or obesity, and diabetes^21^. Taken together, these findings suggest that in individuas with psychosis, ChT is associated with rumination, negative schemas, poor diet, and reduced physical activity, which can be grouped as a “negative life style”.

Our first aim was to understand how from a network perspective, ChT and negative life style interact in a given network and what are the central and bridge symptoms. The second aim was to determine whether the network characteristics of the two domains would remain the same or be altered after including positive (P) and negative symptoms (N) of psychosis as a third domain in the network. Therefore, in the present study, networks consisting of two or three domains were estimated and analyzed in patients with early-stage psychosis. The characteristics of these networks were compared to those of healthy controls.

## Method

### Study sample

Data were collected as part of the longitudinal multicenter Korean Early Psychosis Study (KEPS), which has been described in detail elsewhere^22^. The sample comprised 500 patients with early-stage psychosis and 202 healthy controls. The inclusion criteria required that subjects be between 19 and 58 years of age and meet the Diagnostic and Statistical Manual of Mental Disorders, Fourth Edition (DSM-IV)^23^ criteria for schizophrenia spectrum disorders (schizophrenia, schizoaffective disorder, schizophreniform disorder, psychotic disorder not otherwise specified [NOS]), brief psychotic disorder, or delusional disorder. Individuals who had been treated with antipsychotics for < 2 years were considered to be in early-stage psychosis. Written informed consents were obtained from all the participants and also from legal guardians of the participants. All experimental protocols were approved by the Ethics Committee of the Jeonbuk National University Hospital (approval number CUH 2014-11-002). All procedures were performed in accordance with relevant guidelines.

### Measures

The severity of psychiatric symptoms was assessed using the Positive and Negative Syndrome Scale (PANSS)^24^. For self-rating scales, the Brief Core Schema Scale (BCSS)^15^, Brooding Scale (BS)^25^, Early Trauma Inventory Self Report-Short Form (ETI)^26^, Dietary Habits Questionnaire (DHQ)^27^ and Physical Activity Rating (PAR)^28^ were employed. The DHQ is a 20-item self-administered questionnaire consisting of three subcategories: five items for diet regularity, six items for balanced diet, and nine items for unhealthy diet and eating habits. This scale was developed based on dietary guidance published by the Korean Ministry for Health, Welfare and Family Affairs (2010)^29^. The total score is categorized as indicating poor (20–49), usual (50–79), or good (80–100) diet. The PAR is a questionnaire that rates the individual’s level of physical activity, with scores ranging from 0 (avoids walking or exercise) to 7 (runs more than 10 miles per week or spends more than 3 h per week in comparable physical activity). As all scores for each parameter exhibited skewed distributions based on the Shapiro–Wilk test, they were normalized using nonparanormal transformation^30,31^.

### Network estimation

Networks were constructed using 12 nodes: negative self, positive self, negative others, and positive others from the BCSS; emotional and cognitive components from the BS; DHQ; PAR; and general trauma, emotional abuse, physical abuse, and sexual abuse from the ETI). We fitted a Gaussian graphical model (GGM) to the data. The GGM networks were regularized via a graphical lasso (GLASSO) algorithm^32^ in combination with the extended Bayesian information criterion (EBIC) model. A tuning hyperparameter γ for the EBIC was set to 0.5^33^. The edges were calculated by partial correlations. We used the R-packages ‘bootnet (estimateNetwork (https://CRAN.R-project.org/package=bootnet. R package version 1.4.3))’ and ‘qgraph’ (https://rdocumentation.org/packages/qgraph/versions/1.6.9. R package version 1.6.9) to estimate and visualize all networks^34^.

### Network analysis

#### Global network metrics

Global network metrics consisting of network density, global strength, averaged clustering coefficient, modularity index (Q), and characteristic path length were calculated using the R packages ‘qgraph’ and ‘igraph’ (https://cran.r-project.org/package=igraph. R package version 1.2.7).

#### Local network metrics

Although strength is regarded as the most reliably estimated centrality index it does not necessarily indicate the degree to which a node can be predicted by the remaining intranetwork nodes. To examine node predictability, we estimated the proportion of each node’s variance accounted for by its connections to other nodes in the network, using the ‘mgm’ package (https://cran.r-project.org/package=mgm. R package version 1.2-12). In addition, as strength centrality uses the sum of absolute weights, whether positive or negative, which might distort interpretation, we estimated expected influence (EI), i.e., the sum of all edges of a node^35^. To detect symptoms that bridged the two domains (ChT and negative life style) or three domains (ChT, negative life style and P and N on the PANSS), bridge EI was calculated. Bridge EI is the sum of the values (+ or −) of all edges that connect a node to all nodes that are not part of the same community^36^. Bridge symptoms that play a primary role in connecting two or more psychiatric symptoms or domains^37^ were defined as those items scoring higher than the 80th percentile for the bridge EI metric. We also computed the shortest pathways^38^ from each subscale of the ETI to negative life style or to P and N within the network. To determine the EI, bridge EI, and shortest pathway, the R-packages ‘mgm’, ‘qgraph’, ‘networktools’^39^, and ‘igraph’ were used, respectively.

### Network comparison

We investigated network structures and global strength using the Network Comparison Test (NCT) in the R package. For global network metrics (network density, averaged clustering coefficient, modularity index [Q], and characteristic path length), the ‘NetworkToolbox’ package (https://cran.r-project.org/package=NetworkToolbox. R package version 1.4.2) was used to explore whether the overall level of network connectivity was equal among the networks.

### Network accuracy and stability

The accuracy and stability of the network were examined using the R package ‘bootnet(https://CRAN.R-project.org/package=bootnet. R package version 1.4.3)’. First, we bootstrapped (1,000 iterations) the 95% confidence intervals around the edge weights to assess the accuracy of the edge weights. Second, we used the case-dropping subset bootstrap (1,000 iterations) to examine the stability of the order of the node centrality indices. A correlation stability coefficient (CS-coefficient), a measure that quantifies the stability of node centrality indices, was also calculated. Finally, we tested for significant differences in edge weights and node centralities using the bootstrapped difference tests. In addition, using the R-package “netPower (https://github.com/mihaiconstantin/netpaw. R package version 1.0.0)”, we calculated power with current conditions which was 80%, an acceptable level.

## Results

### Participants’ demographic and clinical characteristics

The proportion of males was lower (*p* = 0.027) and the mean age was younger (*p* < 0.001) in patients compared to controls. The scores on the negative self and negative others of the BCSS, the emotional and cognitive components on the BS, the general trauma, emotional abuse, physical abuse, and sexual abuse on the ETI, and the DHQ were significantly higher in patients compared to controls. Notably, although the DHQ score was significantly higher in patients, scores ranging from 50 to 79 are categorized as usual; hence, there was no actual difference between the two groups. The PAR score was significantly lower in patients (Table S1).

### Global network metrics

Comparisons between the two groups revealed no significant differences in all global network metrics except the average clustering coefficient (Table S2).

### Local network metrics

**Figure 1:**
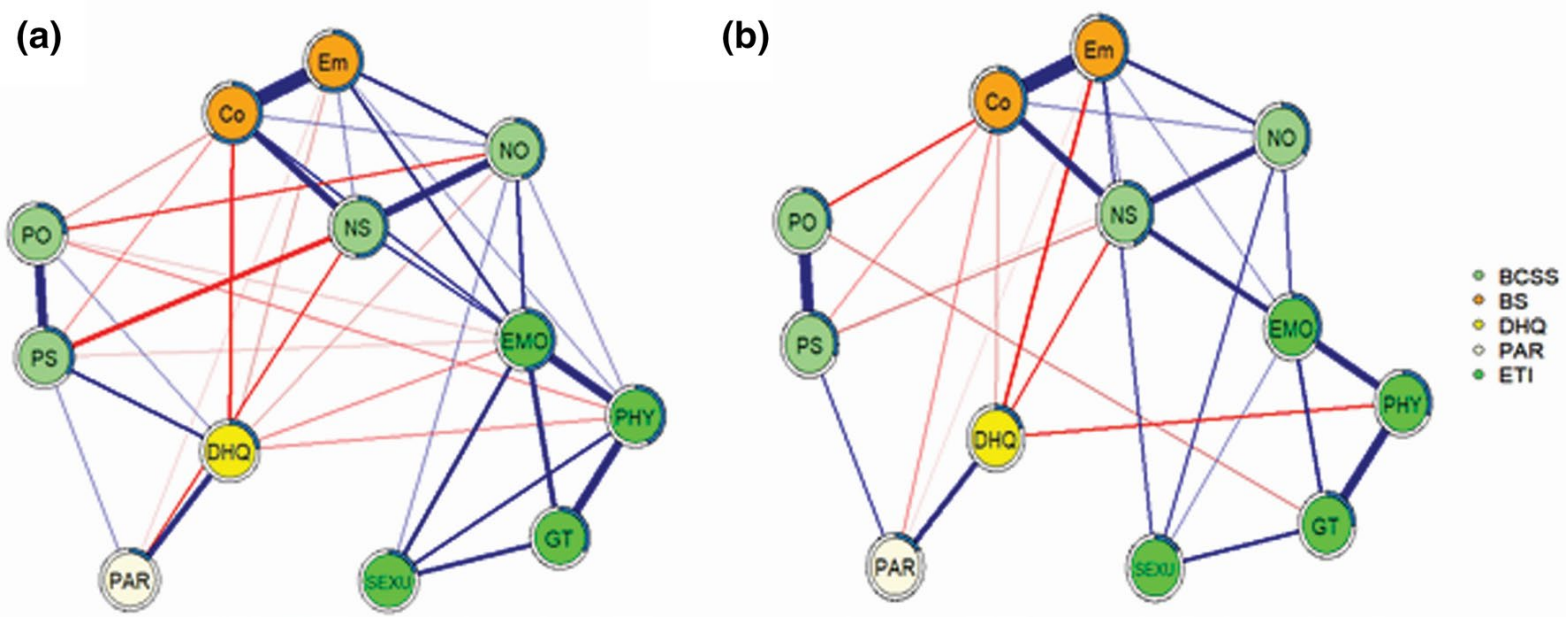
Estimated network structures of (**a**) patients and (**b**) controls. Graph features represent the following: edge thickness = strengths of the regularized partial correlations (positive in blue and negative in red); shaded area in the node perimeter = predictability. Abbreviation: BCSS, Brief Core Schema Scales; BS, Brooding Scale; Co, Cognitive subscale of the BS; DHQ, Dietary Habits Questionnaire; Em, Emotional subscale of the BS; EMO, Emotional abuse of the ETI; ETI, Early Trauma Inventory Self Report-Short Form; GT, General Traumatic experiences of the ETI; NO, Negative-Others of the BCSS; NS, Negative-Self of the BCSS; PAR, Physical Activity Rating; PHY, Physical abuse of the ETI; PO, Positive-Others of the BCSS; PS, Positive-Self of the BCSS; SEXU, Sexual abuse of the ETI. The R-packages ‘mgm (version 1.2-12 and URL https://CRAN.R-project.org/package=mgm)’ ‘qgraph (version 1.6.9 and URL https://CRAN.R-project.org/package=qgraph)’ and ‘igraph (version 1.2.7 and URL https://CRAN.R-project.org/package=igraph)’ were used to estimate and visualize all networks.

**Figure 2:**
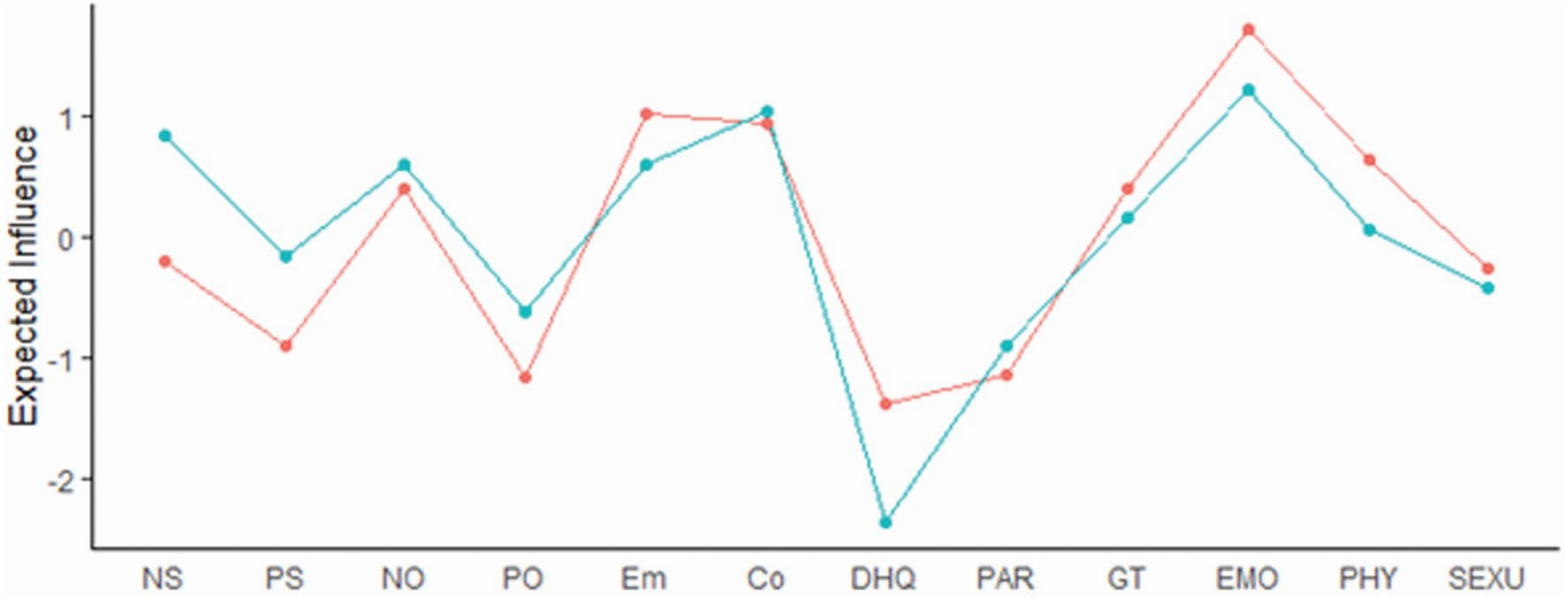
Expected influence for patients (red) and controls (blue). Abbreviation: Co, Cognitive subscale of the BS; DHQ, Dietary Habits Questionnaire; Em, Emotional subscale of the BS; EMO, Emotional abuse of the ETI; GT, General Traumatic experiences of the ETI; NO, Negative-Others of the BCSS; NS, Negative-Self of the BCSS; PAR, Physical Activity Rating; PHY, Physical abuse of the ETI; PO, Positive-Others of the BCSS; PS, Positive-Self of the BCSS; SEXU, Sexual abuse of the ETI The R-packages ‘mgm (version 1.2-12 and URL https://CRAN.R-project.org/package=mgm)’ and ‘qgraph (version 1.6.9 and URL https://CRAN.R-project.org/package=qgraph)’ were used.

**Figure 3:**
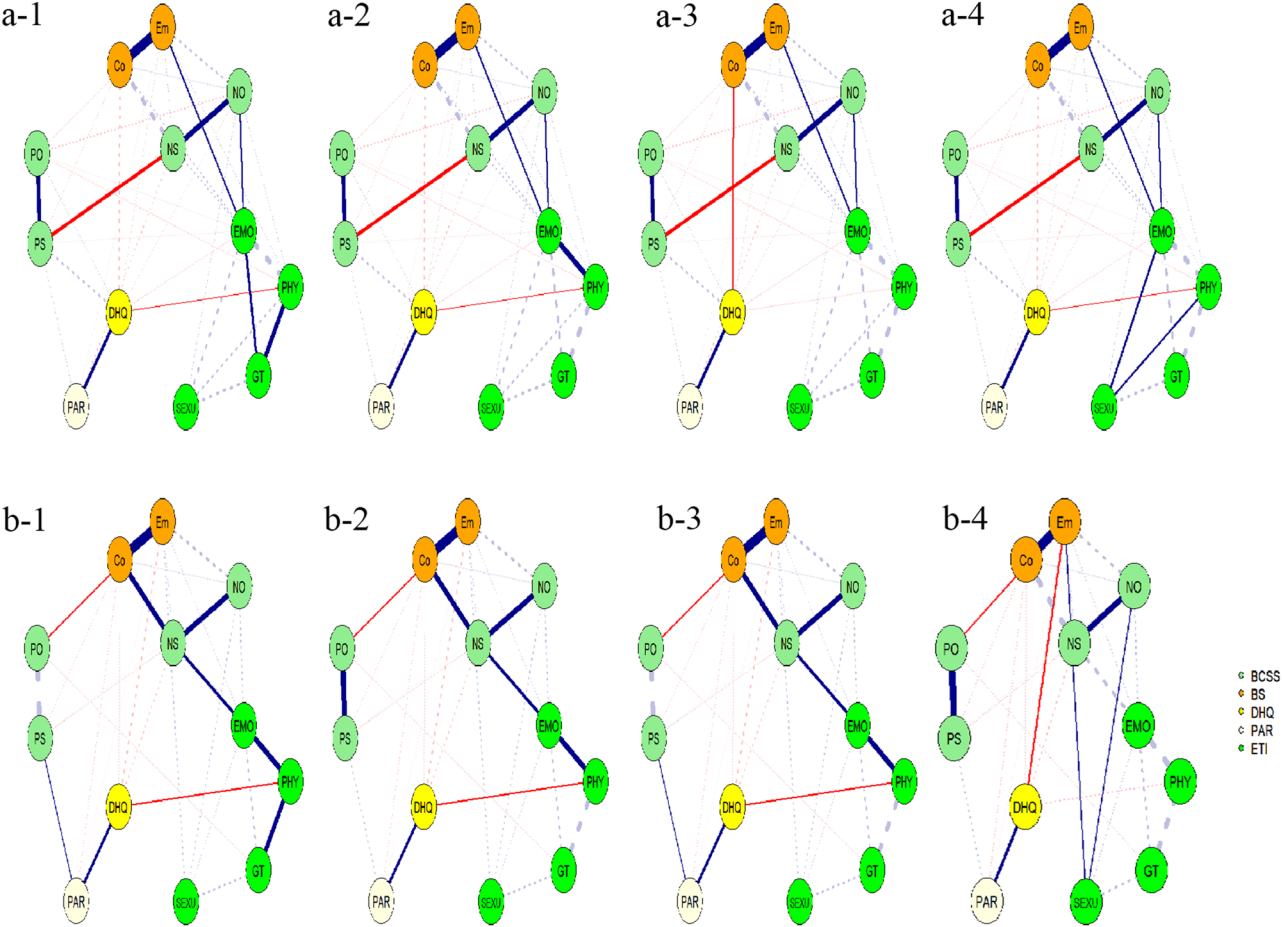
Shortest pathways from GT, EMO, PHY or SEXU to negative life style in (**a**) patients (**a-1**, **a-2**, **a-3** and **a-4**) and (**b**) controls (**b-1**, **b-2**, **b-3** and **b-4**). Thicker solid lines represent stronger connections; Dashed lines represent background connections existent within the network that are less relevant when investigating shortest paths. Abbreviation: same as in the Fig. 1. The R-packages ‘networktools (version 1.4.0 and URL https://CRAN.R-project.org/package=networktools)’ and ‘igraph (version 1.2.7 and URL https://CRAN.R-project.org/package=igraph)’ were used.

**Figure 4:**
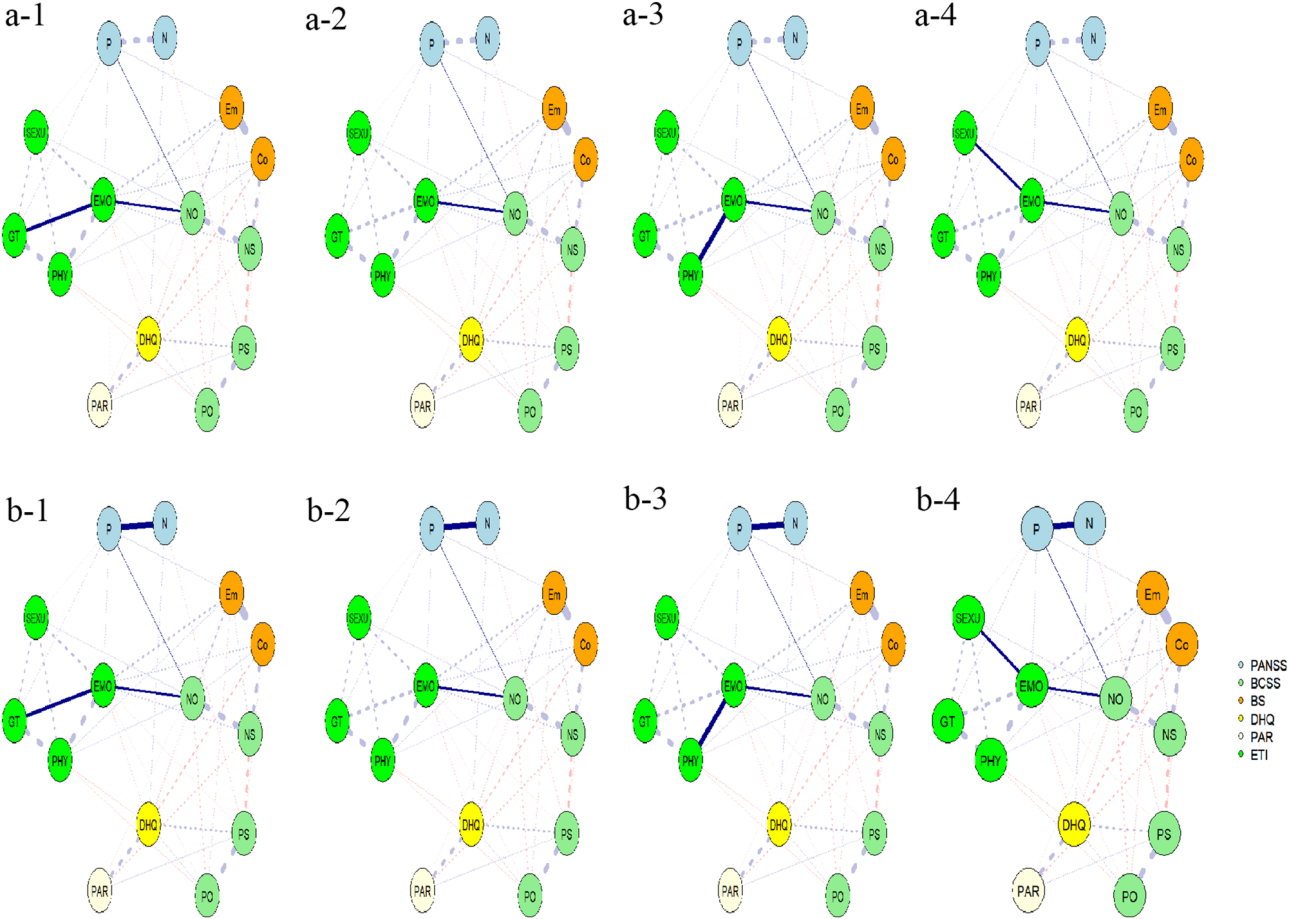
Shortest pathways from GT, EMO, PHY or SEXU to P (**a-1**, **a-2**, **a-3** and **a-4**) or N (**b-1**, **b-2**, **b-3** and **b-4**) in patients. Thicker solid lines represent stronger connections; Dashed lines represent background connections existent within the network that are less relevant when investigating shortest paths. Abbreviation: same as in the Fig. 1. The R-packages ‘networktools (version 1.4.0 and URL https://CRAN.R-project.org/package=networktools)’ and ‘igraph (version 1.2.7 and URL https://CRAN.R-project.org/package=igraph)’ were used.

**Figure 5:**
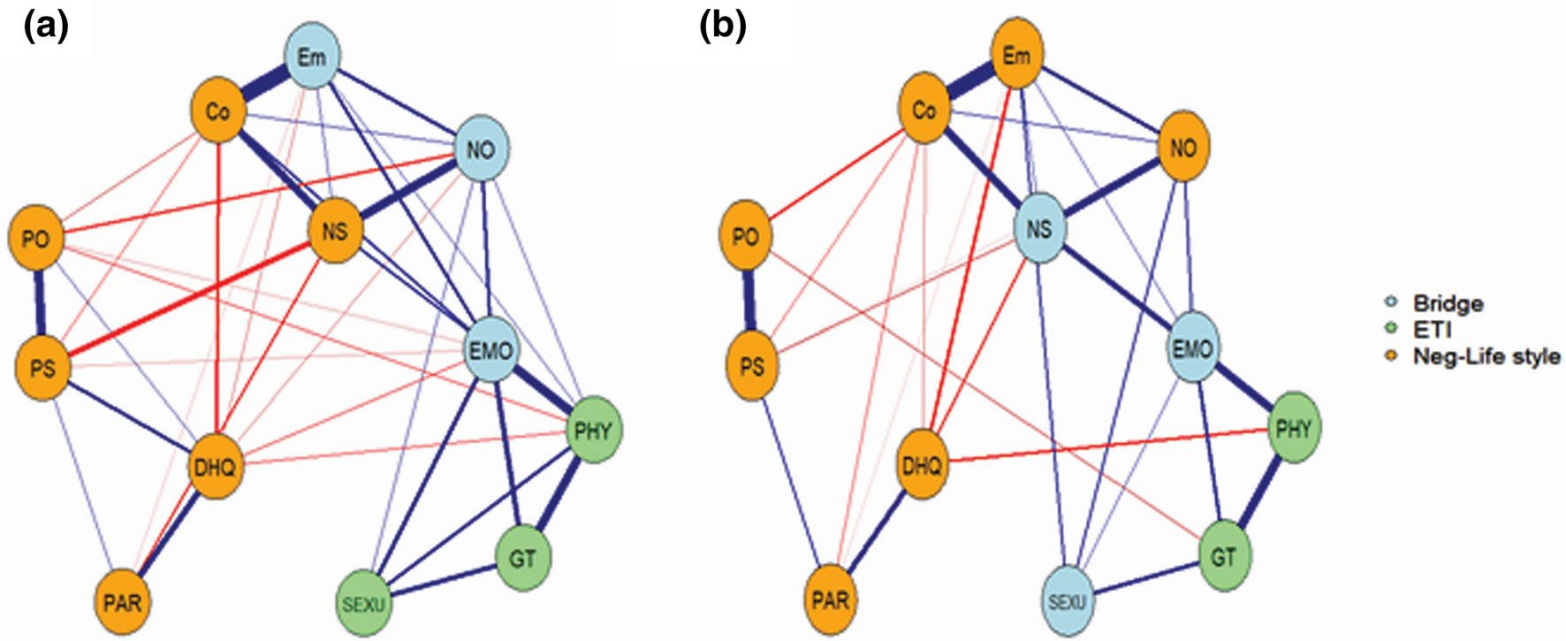
Bridge symptoms between two domains (childhood trauma and negative life style) in (**a**) patients and (**b**) controls. Abbreviation: same as in the Fig. 1. The R-packages ‘mgm (version 1.2–12 and URL https://CRAN.R-project.org/package=mgm)’ and ‘qgraph (version 1.6.9 and URL https://CRAN.R-project.org/package=qgraph)’ were used.

The nodes showing the highest predictability in both groups were cognitive and emotional components of the BS (Figs. 1 and S1 and Table S3). The node with highest EI in both groups was emotional abuse of the ETI (Figs. 2 and S2). In patients, the shortest pathway from each subscale node of the ETI to the BS nodes always connected through the emotional abuse node (Fig. 3a). However, in controls, it always connected via the negative self and/or emotional abuse nodes. In patients, the connection order for the BS nodes was emotional and cognitive components, whereas it was the reverse in controls. With respect to the shortest pathway from each subscale node of the ETI to the BCSS nodes, emotional abuse played the same mediating role in both groups (except for sexual abuse in the controls, which connected directly to negative self). However, the connection order for the BCSS nodes was negative others and negative self in patients and the reverse in controls (Fig. 3b). When P and N of the PANSS were included in the network, the shortest pathway from each subscale node of the ETI to the P or N node always connected through the negative others node (Fig. 4). The bridge symptoms between the two domains were emotional abuse, negative others and emotional component in patients and emotional abuse, negative self and sexual abuse in controls (Fig. 5). The bridge symptoms among the three domains in patients were the same as in the two-domain model (Fig. S3).

### Network accuracy and stability

The results of the edge weight bootstrap analysis (Fig. S4) showed substantial overlap among the 95% CIs of the edge weights. However, some of the strongest edges showed non-overlapping CI values. The CS coefficients for node strength and bridge EI were $\ge$ 0.25 in both groups (Table S4 and S5). The results of bootstrapped difference tests for EI, bridge EI, edge weights and strength centralities are presented in Figs. S1–S9.

## Discussion

This childhood adversity can affect many aspects of personal development. Assuming that ChT may lead to negative life style factors such as rumination, negative schemas, poor diet, and reduced physical exercise, we conducted network analysis to explore dynamic interactions between ChT and negative life style in individuals with early-stage psychosis. Our results revealed several central symptoms within a network, which could ultimately prove to be important targets for clinical intervention.

In terms of global network metrics, only the average clustering coefficient differed significantly between the patient and control groups. As a higher clustering coefficient value means a more connected neighborhood around one particular node, this suggests that the 12 symptoms studied in patients were much more highly connected in a triangular fashion, producing greater negative impact on one another. Given that clustering coefficients tend to decline with the age^40^, our finding may be due to the age difference between the groups. However, no test controlling for covariates is available at present.

With respect to local network metrics, in both groups, the node with the highest predictability was rumination, which indicates that variance in rumination was highly predicted by its relationships with other symptoms in the network. Numerous factors may trigger rumination including negative affect, childhood adversity^41^, stable individual traits, and failure to achieve a goal^42^. Rumination has also been regarded as a common pathway leading to the development of mental disorders^43^. emotional abuse was the most influential central symptom in both groups, although its EI value was slightly higher in patients compared to controls. Emotional abuse and emotional neglect have been considered the most frequent forms of severe trauma in patients with schizophrenia^44^. Few studies have addressed the specificity of childhood adversity effects; emotional and physical abuses were correlated with dissociative symptoms in patients with schizophrenia^44^; emotional neglect was associated with psychotic experiences in a general population cohort^45^; and physical and sexual abuses were associated with positive symptoms in first-episode psychosis^46^. Therefore, these findings suggest that, from the perspective of “factors that are influenced and influential”, rumination and emotional abuse are key central factors that should be targeted for psychosocial intervention in patients with psychosis.

With regard to the shortest pathway between ChT and negative life style, the results suggest that emotional abuse played the same mediating role between the two in both groups. However, a key difference was that in controls, this pathway always led through negative self following emotional abuse and then connected to rumination or negative others. More importantly, in the network including P and N, the pathway for patients was always through negative others following emotional abuse and was then connected to P or N. Thus, these findings suggest that, although emotional abuse was an important meditator in both groups, negative others and negative self were more crucial as differentiating meditators for patients and controls, respectively. Several studies have demonstrated a mediating role of negative beliefs about others between trauma and psychotic symptoms^17^ and a close association of negative views of others with paranoia^47^. It is noteworthy that the ‘negative self’ schema is also closely associated with persecutory delusions^48^ and auditory verbal hallucinations^49^. In a community sample, previous evidence indicated that early trauma led to reduced self-esteem^50^ or negative self-referential processing^51^. Taken together, our findings suggest that patients with early-stage psychosis may benefit from psychotherapeutic treatment targeting negative others. One systematic review of schema therapy indicated initial significant results in terms of reducing early maladaptive schemas and improving symptoms related to personality disorders, but evidence for other mental disorders is currently sparse^52^. The question of why negative others or negative self was a more important meditator in patients than in controls needs to be clarified in future studies. The relative importance of negative others vs. negative self in patients vs. controls was supported by the findings about bridge symptoms. Interestingly, when P and N were included in the network, three bridge symptoms (emotional abuse, negative others, and emotional component of the BS) remained the same. As bridge symptoms connect different domains within a network, the activation of these bridge symptoms might be expected to distribute the activation toward other domains. This emphasizes the importance of intervening in negative others to reduce P and N in patients with psychosis. Importantly, the CS coefficients for node strength and bridge EI were above the recommended 0.25 cutoff in both groups, making their interpretation acceptable.

A few limitations associated with the current study need to be acknowledged. First, we used cross-sectional data to examine the association between ChT and negative life style, which did not allow us to identify potentially causal relations. Second, sex covariate was not controlled for. Given high impact of sexual abuse in female, our results should be limitedly interpreted. Third, as ChT was assessed with self-rating scale, its validity is weak. Lastly, to validate our findings, replication study should be pursued in future. Despite these caveats, the strength of our study lay in the use of a network approach to examine interrelationships between ChT and negative life style in patients and controls. In summary, we found that rumination and emotional abuse were central symptoms in both groups, and negative others and negative self played important mediating roles for patients and controls, respectively. These findings highlight the importance of targeting central or mediating symptoms to improve patients’ recovery. Future research focusing specifically on these symptoms could be invaluable when designing interventions for patients exposed to ChT.

## Supplementary Information

Supplementary Information.

## Data Availability

The data supporting the findings are fully available without restriction. Relevant data are available from the corresponding author upon request.
