# Supplementary material for: Network analysis of trauma in patients with early-stage psychosis
Source: Sci Rep. 2021 Nov 23;11:22749. doi: 10.1038/s41598-021-01574-y (PMC8610987; doi:10.1038/s41598-021-01574-y)
Supplement: Supplementary file 1 — Supplementary Information. [file 41598_2021_1574_MOESM1_ESM.pdf]

Network Analysis of Trauma in Patients

with Early-stage Psychosis

Young Chul Chung MD, PhD*[,1,2,3], Je-Yeon Yun MD, PhD[4,5], Thong Ba Nguyen PhD[1,2], Fatima Zahra Rami MS[1,2], Yan Hong Piao MD, PhD[1,2], Ling Li MD[1,2], Bo Mi Lee BA[1,2], Woo-Sung Kim MR[1,2], Jing Sui MD, PhD[6,7], Sung-Wan Kim MD, PhD[8], Bong Ju Lee MD, PhD[9], Jung Jin Kim MD, PhD[10], Je-Chun Yu MD[11], Kyu Young Lee MD, PhD[12], Seung-Hee Won MD, PhD[13], Seung-Hwan Lee MD, PhD[14], Seung-Hyun Kim MD, PhD[15], Shi Hyun Kang MD, PhD[16], Eui Tae Kim MD, PhD[17]

[1]Department of Psychiatry, Jeonbuk National University Medical School, Jeonju, Republic of Korea, 54907.

[2]Research Institute of Clinical Medicine of Jeonbuk National University-Biomedical Research Institute of Jeonbuk National University Hospital, Jeonju, Republic of Korea, 54907.

[3]Department of Psychiatry, Jeonbuk National University Hospital, Jeonju, Republic of Korea, 54907.

[4]Seoul National University Hospital, Seoul, Republic of Korea, 03080.

[5]Yeongeon Student Support Center, Seoul National University College of Medicine, Seoul, Republic of Korea, 03080.

[6]Brainnetome Center and National Laboratory of Pattern Recognition, Institute of Automation, Chinese Academy of Sciences, Beijing, China, 100190.

[7]University of Chinese Academy of Sciences; CAS Center for Excellence in Brain Science and Intelligence Technology, Chinese Academy of Sciences, Beijing, China, 100049.

[8]Department of Psychiatry, Chonnam National University Medical School, Gwangju, Republic of Korea, 501-746.

[9]Department of Psychiatry, Inje University Haeundae Paik Hospital, Inje University College of Medicine, Busan, Republic of Korea, 48108.

[10]Department of Psychiatry, The Catholic University of Korea, Seoul St. Mary's Hospital, Seoul, Republic of Korea, 705-718.

[11]Department of Psychiatry, Eulji University School of Medicine, Eulji University Hospital, Daejeon, Republic of Korea, 34824.

[12]Department of Psychiatry, Eulji University School of Medicine, Eulji General Hospital, Seoul, Republic of Korea, 34824.

[13]Department of Psychiatry, Kyungpook National University School of Medicine, Daegu, Republic of Korea, 41944.

[14]Department of Psychiatry, Inje University College of Medicine, Goyang, Republic of Korea, 50834.

[15]Department of Psychiatry, Korea University College of Medicine, Guro Hospital, Seoul, Republic of Korea, 136-701.

[16]Department of Psychiatry, Seoul National Hospital, Seoul, Republic of Korea, 463-707.

[17]Department of Psychiatry, Seoul National University Bundang Hospital, Seongnam, Republic of Korea, 463-707.

*Correspondence to: Dr Young Chul Chung, Department of Psychiatry, Chonbuk National University Medical School, Keumam Dong 634-18, Jeonju 561-712, Republic of Korea; tel: +82-63-250-2185, fax: +82-63-275-3157, e-mail: chungyc@jbnu.ac.kr (Dr Young Chul Chung)

Table S1. Correlation stability coefficient of centrality indices

**Centralities**	**Patients**	**Controls**
Strength	0.749	0.441
Closeness	0.594	0.05
Betweenness	0.238	0.05
Bridge expected influence	0.749	0.752

Table S2. **Predictability estimates**

Node	RMSE		R^2	
	Patients	Controls	Patients	Controls
NS	0.697	0.747	0.513	0.44
PS	0.808	0.83	0.346	0.308
NO	0.739	0.807	0.453	0.345
PO	0.864	0.84	0.251	0.29
Em	0.649	0.69	0.578	0.522
Co	0.618	0.671	0.617	0.547
DHQ	0.873	0.893	0.236	0.199
GT	0.82	0.855	0.326	0.265
EMO	0.705	0.814	0.502	0.335
PHY	0.756	0.831	0.427	0.307
SEXU	0.895	0.919	0.197	0.152
PAR	0.93	0.928	0.132	0.135
Mean	0.779	0.818	0.381	0.320

Abbreviation: Co, Cognitive subscale of the BS; DHQ, Dietary Habits Questionnaire; Em, Emotional subscale of the BS; EMO, Emotional abuse of the ETI; GT, General Traumatic experiences of the ETI; NO, Negative-Others of the BCSS; NS, Negative-Self of the BCSS; PAR, Physical Activity Rating; PHY, Physical abuse of the ETI; PO, Positive-Others of the BCSS; PS, Positive-Self of the BCSS; SEXU, Sexual abuse of the ETI.

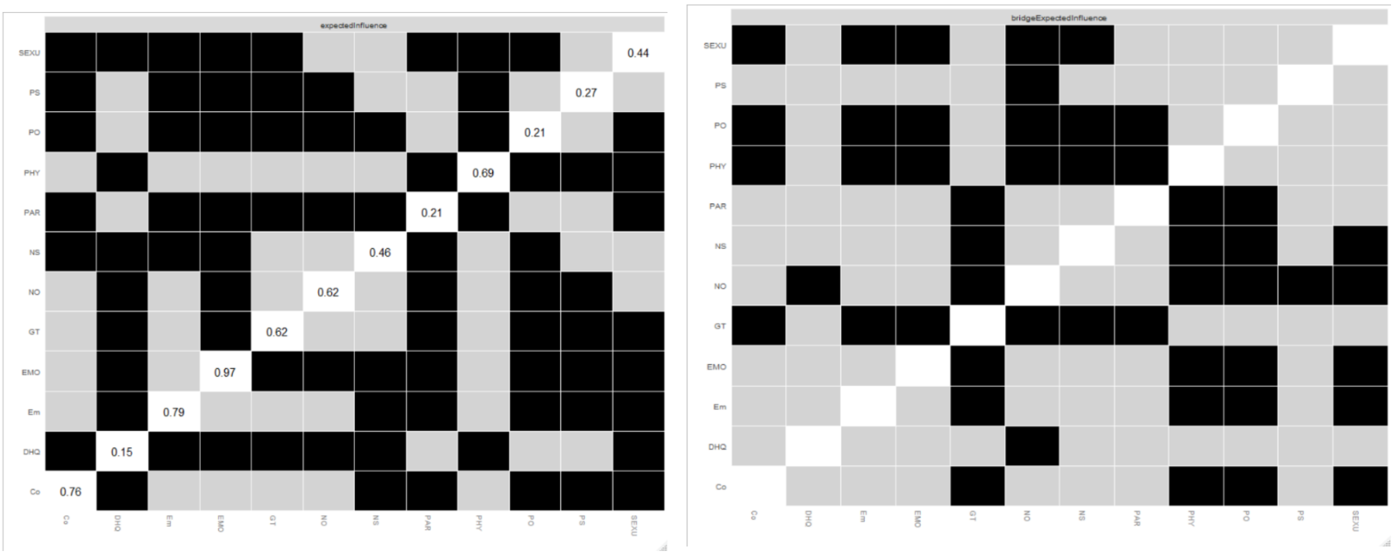

Figure S1. Bootstrapped difference tests for a) node expected influence and b) bridge expected Influence in patients.

Black represents a significant difference for each pairing and grey a non-significant difference.

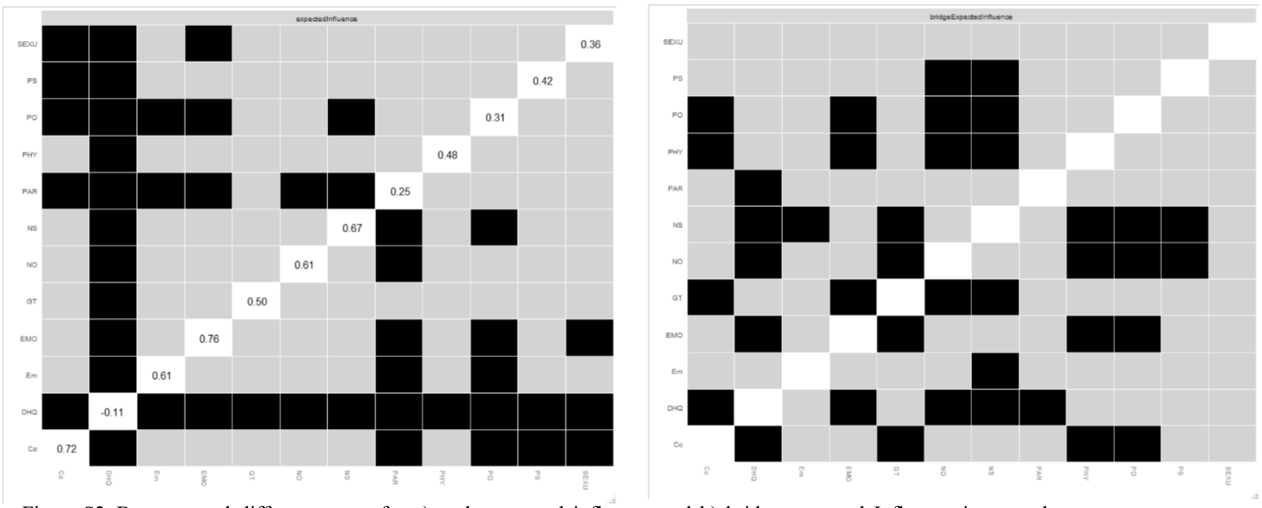

Figure S2. Bootstrapped difference tests for a) node expected influence and b) bridge expected Influence in controls.

Black represents a significant difference for each pairing and grey a non-significant difference.

a)

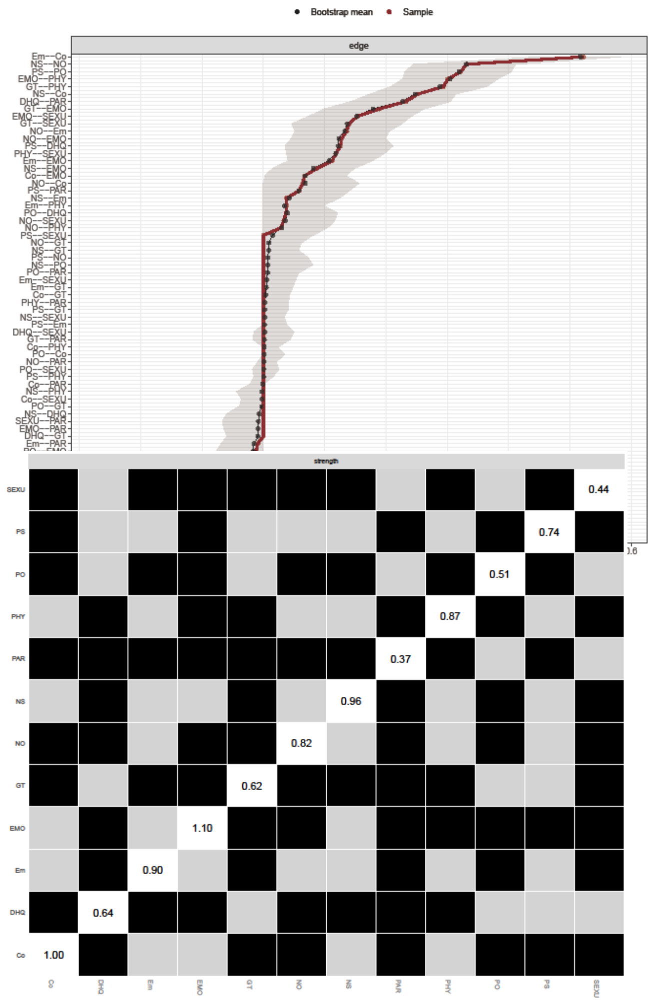

b)

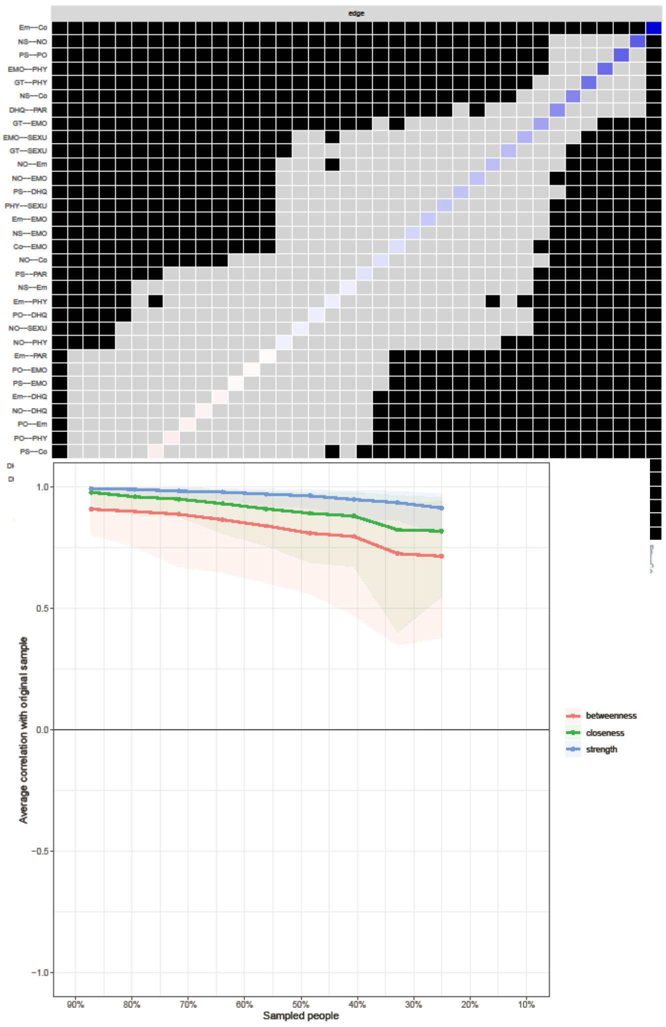

Figure S3. Stability and accuracy in patients.

a) Bootstrapped 95% confidence intervals (CI) for the estimated edge weights in the networks. The red line indicates the sample values and the gray area the bootstrapped CIs. Each horizontal line represents one edge of the network, ordered from the edge with the highest edge-weight to the edge with the lowest edge-weight; b) Bootstrapped difference tests of edge weights in the networks. Black boxes indicate a significant difference between two edges (alpha = .05). Grey boxes indicate no significant difference; c) Bootstrapped difference tests of strength centrality in the networks. Black boxes indicate a significant difference between two edges (alpha = .05). Grey boxes indicate no significant difference; and d) Case-dropping bootstrap for the networks.

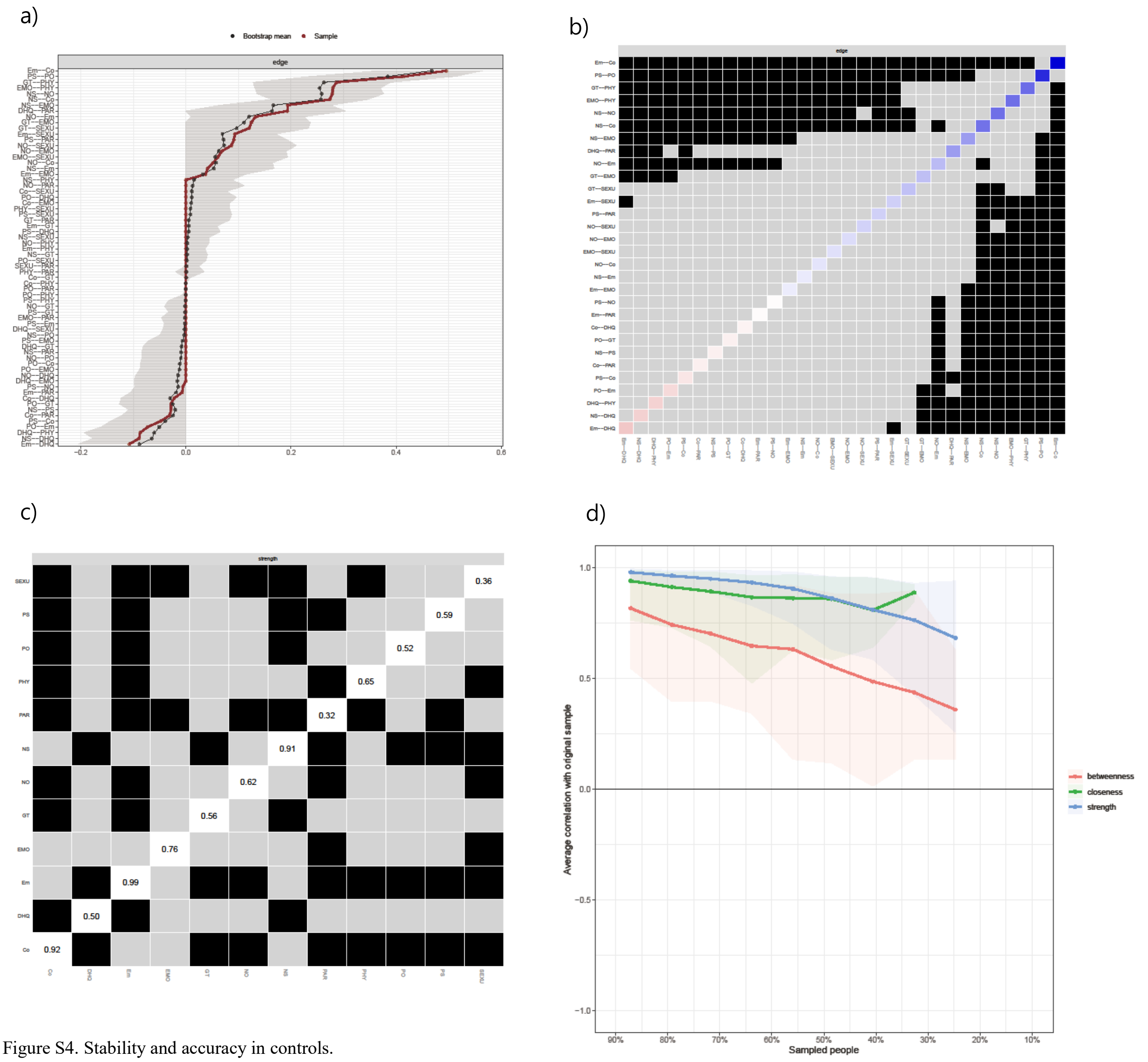

Figure S4. Stability and accuracy in controls.

a) Bootstrapped 95% confidence intervals (CI) for the estimated edge weights in the networks. The red line indicates the sample values and the gray area the bootstrapped CIs. Each horizontal line represents one edge of the network, ordered from the edge with the highest edge-weight to the edge with the lowest edge-weight; b) Bootstrapped difference tests of edge weights in the networks. Black boxes indicate a significant difference between two edges (alpha = .05). Grey boxes indicate no significant difference; c) Bootstrapped difference tests of strength centrality in the networks. Black boxes indicate a significant difference between two edges (alpha = .05). Grey boxes indicate no significant difference; and d) Case-dropping bootstrap for the networks.

Table S3. Global connectivity of the networks including positive and negative symptoms in patients

Characteristics	Network
Density	0.505
Average shortest pathlength	1.516
Average clustering coefficient	0.620
Q Modularity	0.408

Table S4. Correlation stability coefficient of centrality indices

Centralities	Patients	Controls
Strength	0.749	0.441
Closeness	0.594	0.05
Betweenness	0.238	0.05
Bridge expected influence	0.749	0.752

Table S5. Correlation stability coefficient of centrality indices of the networks including positive and negative symptoms in patients

Centralities	Network
Strength	0.749
Closeness	0.516
Betweenness	0.05
Bridge EI	0.749

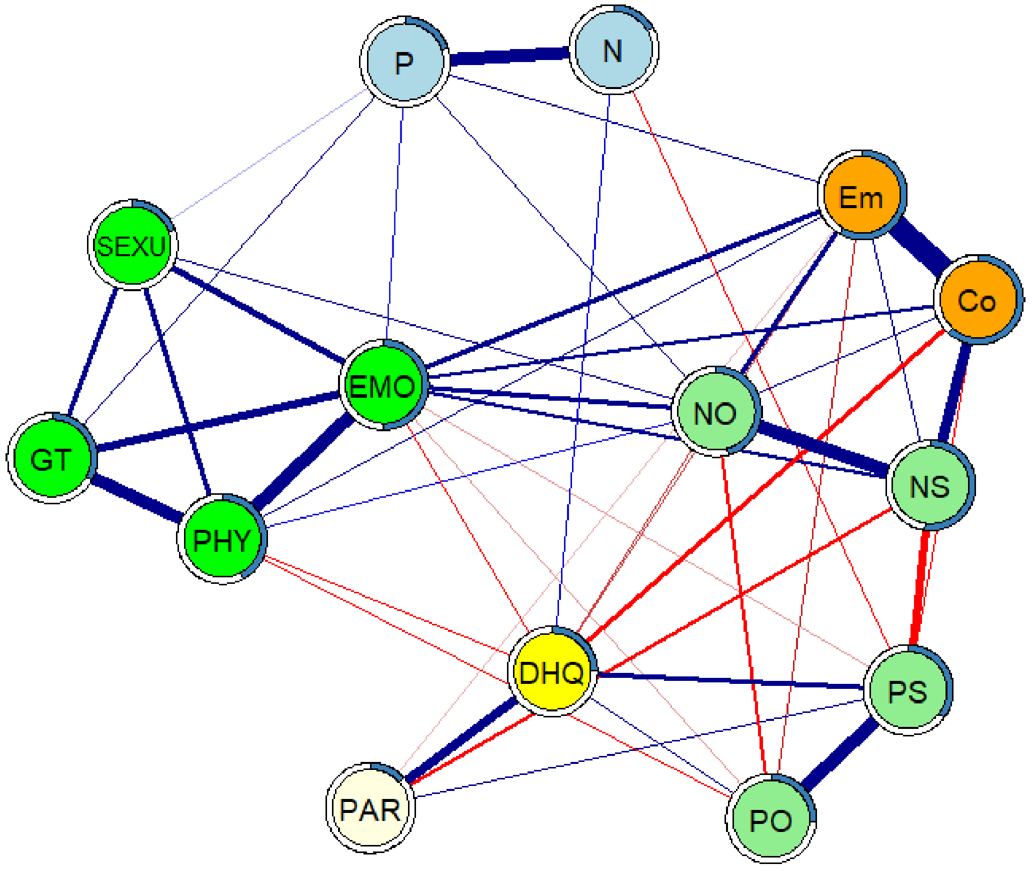

Figure S5. Estimated network structure including positive and negative symptoms in patients.

Graph features represent the following: edge thickness = strengths of the regularized partial correlations (positive in blue and negative in red); shaded area in the node perimeter=predictability.

Abbreviation: same as in the table S2

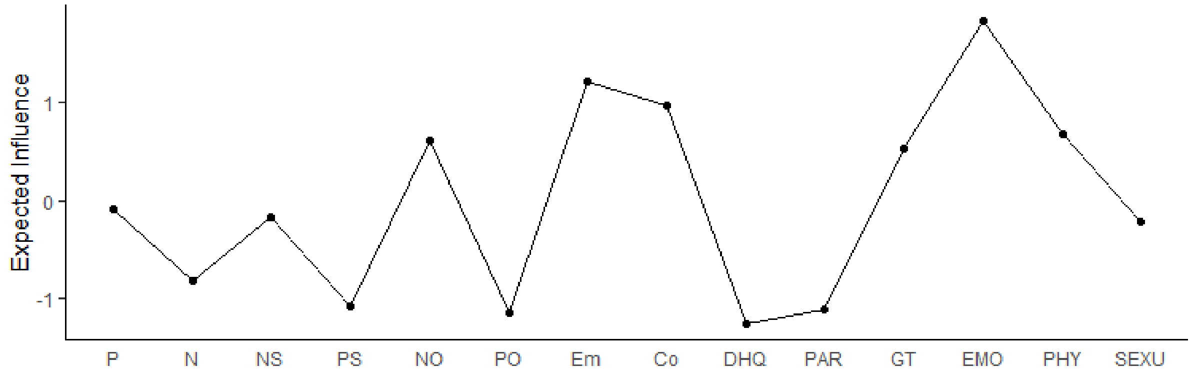

Figure S6. Expected influence of the network including positive and negative symptoms in patients.

Abbreviation: same as in the table S2

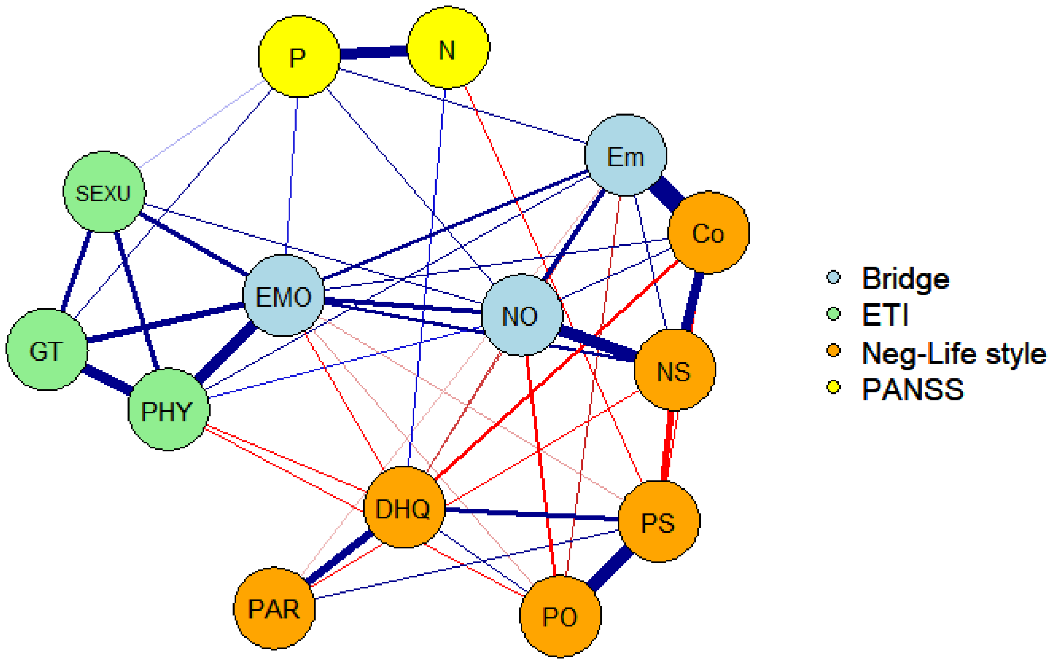

Figure S7. Bridge symptoms among three domains (childhood trauma, negative life style and P and N) in patients.

Abbreviation: ETI, Early Trauma Inventory Self Report-Short Form; Neg-Life Style includes Co, Em, NO, NS, PO, PS, DHQ and PAR; PANSS, Positive and Negative Syndrome Scale; P, Positive symptoms; N, Negative symptoms.

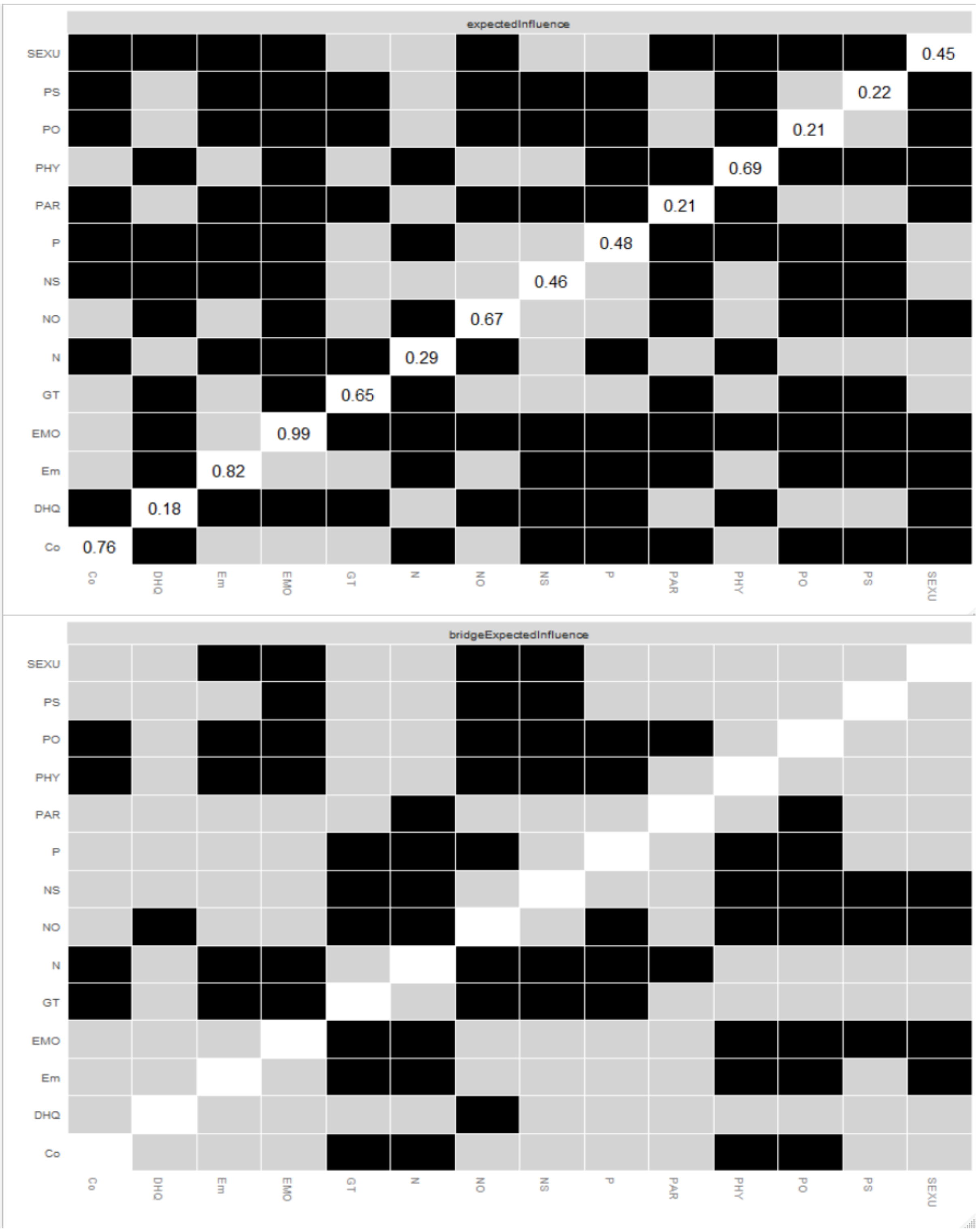

Figure S8. Bootstrapped difference tests for a) node expected influence and b) bridge expected influence in patients. Black represents a significant difference for each pairing and grey a non-significant difference.

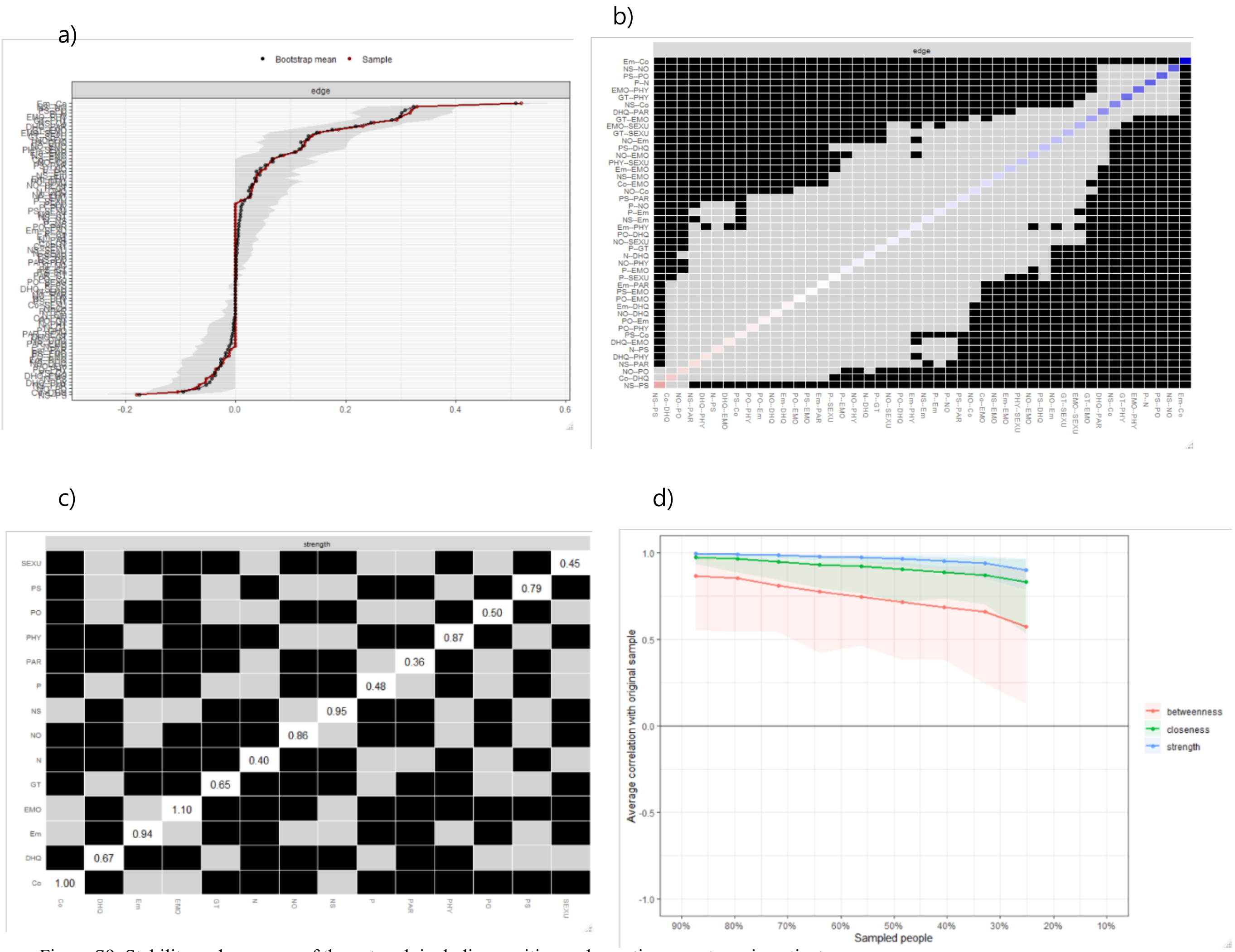

Figure S9. Stability and accuracy of the network including positive and negative symptoms in patients.

a) Bootstrapped 95% confidence intervals (CI) for the estimated edge weights in the networks. The red line indicates the sample values and the gray area the bootstrapped CIs. Each horizontal line represents one edge of the network, ordered from the edge with the highest edge-weight to the edge with the lowest edge-weight; b) Bootstrapped difference tests of edge weights in the networks. Black boxes indicate a significant difference between two edges (alpha = .05). Grey boxes indicate no significant difference; c) Bootstrapped difference tests of strength centrality in the networks. Black boxes indicate a significant difference between two edges (alpha = .05). Grey boxes indicate no significant difference; and d) Case-dropping bootstrap for the networks.

```
# R-Codes
##########################################################################
## Install the networktools package
#install.packages("networktools")
#require("networktools")
library("readxl")
library(qgraph)
library(huge)
library("psychTools")
library(reshape2)
library(NetworkComparisonTest)
library(bootnet)
library(data.table)
#library("Deducer")
library("igraph")
# Open data:
set.seed(123)
setwd("C:/Users/THONG/Desktop/PROJECT1/network")
data <- read_excel("2020-KEPS raw data-Trauma Network analysis-7.xlsx",sheet = "Raw data")
summary(df1)
#setnames
df=data
setnames(df, old = c("PANSS-P-TO","PANSS-N-TO", "PANSS-G-TO", "CDSS TO",
                    "BCSS-NS","BCSS-PS","BCSS-NO","BCSS-PO","BS-E",   "BS-CO",   "ETI","DHQ",   "PAR1","ETI-GE",   "ETI-EMO","ETI-PHY","ETI-SEXU"),
        new = c("P","N","G", "CS","NS","PS","NO","PO","Em", "Co", "ETI","DHQ", "PAR","GT","EMO", "PHY","SEXU"))
dat=subset(df,Group=="Treatment")
df1= dat[, c("NS","PS","NO","PO","Em", "Co", "DHQ","PAR","GT","EMO","PHY", "SEXU")] # remission group at baseline
dat2=subset(df,Group=="Con")
df2= dat2[, c("NS","PS","NO","PO","Em", "Co", "DHQ","PAR","GT","EMO","PHY", "SEXU")] # remission group at baseline
df3= dat[, c("P","N","NS","PS","NO","PO","Em", "Co","DHQ","PAR", "GT","EMO","PHY", "SEXU")]
df1<- na.omit(df1)
```

```
df2<- na.omit(df2)

df3<- na.omit(df3)

#Nonparanormal Transformation

data1 <- huge.npn(df1)

data2<- huge.npn(df2)

data3 <- huge.npn(df3)

##1. Network estimate

#Now let's use the qgraph package to create an EBICglasso network:

net1 <- estimateNetwork(data1,default = "EBICglasso",corMethod = "cor",tuning = 0.5)

net2 <- estimateNetwork(data2,default = "EBICglasso",corMethod = "cor",tuning = 0.5)

net3 <- estimateNetwork(data3,default = "EBICglasso",corMethod = "cor",tuning = 0.5)

#Plot

L <- averageLayout(net1,net2)

L2 <- averageLayout(net3)

layout(t(1:2))

group.item <- list("BCSS" = 1:4,"BS " = 5:6,"DHQ" = 7,"PAR " = 8,"ETI" = 9:12)

group.item2 <- list("PANSS" = 1:2,"BCSS" = 3:6,"BS " = 7:8,"DHQ" = 9,"PAR " = 10,"ETI"=11:14)

color=c("lightblue", "lightgreen", "orange","yellow","lightyellow","green", "purple","red")

layout(t(1:1))

n1=plot(net1, title="a)", groups = group.item,cut = 0.03,negDashed=F,layout = L,color=color,legend=F)

n2=plot(net2, title="b)", groups = group.item,cut = 0.03,negDashed=F,layout = L,color=color)

n3=plot(net3, title="c)", groups = group.item3,cut = 0.03,negDashed=F,layout = L3,color=color)

#get matrices correlatrion of network

Cent=net1$graph

write.xlsx(Cent, file = "Cor.xlsx",sheet = "Cor1",append = TRUE)

### 6.Centrality    of network

centralityPlot(Centrality = list("PANSS" = net3),

               include=c("Strength","Closeness", "Betweenness","ExpectedInfluence"),

               labels = names(df3),decreasing=T)+ theme(legend.title = element_blank())

#2. Network shortest pathways

layout(t(1:1))

pathways(n3, from = c("GT"), to = c("P"))
```

```
pathways(n3, from = c("EMO"), to = c("P"))

pathways(n3, from = c("PHY"), to = c("P"))

pathways(n3, from = c("SEXU"), to = c("P"))

pathways(n3, from = c("GT"), to = c("N"))

pathways(n3, from = c("EMO"), to = c("N"))

pathways(n3, from = c("PHY"), to = c("N"))

n3=plot(net1, groups = group.item1,cut = 0.03,negDashed=F, layout = L1,color=color1,legend=T)

pathways(n3, from = c("SEXU"), to = c("N"))

## 3.Network Perform permutation test & NCT

#density

g1 = as.igraph(n1, attributes=TRUE)

a=(degree(g))/(vcount(g)-1)

g = as.igraph(n1, attributes=TRUE)

b=(degree(g))/(vcount(g)-1)

perm.t.test(a,b,statistic=c("t","mean"),alternative=c("two.sided"), midp=TRUE, B=1000)

graph.density(g1,loop=FALSE)

#Graph coefficient

transitivity(g1,type = "average")

#ShortestPathLengths

CentW <- centrality(g1)

sp <- CentW$ShortestPathLengths

mean(sp[upper.tri(sp,diag=FALSE)])

# The modularity score of that structure

wc <- walktrap.community(g1) # This is looking for structure in your graph

modularity(wc)

library(NetworkComparisonTest)

nct1<- NCT(net1, net2, it=1000,paired = FALSE,test.edges = TRUE,edges="all",

            test.centrality=TRUE,centrality=c("strength","closeness","betweenness"))

nct1<- NCT(net1, net2, it=1000,paired = FALSE,test.edges = TRUE,edges="all",

            test.centrality=TRUE,centrality=c("expectedInfluence"))

nct1

###4.Bridge of Network
```

```
layout(t(1:1))

my_network = n1

community_structure11 <-c(rep("Neg-Life style",10),rep("ETI", 4))

community_structure=community_structure11

## Now we are ready to look at bridge centrality!

## It's a one-liner with the bridge() function:

require("networktools")

bridge_centrality <- bridge(my_network, communities=community_structure)

bridge_centrality

## It's very easy to get a nice plot of this:

plot(bridge_centrality) ## We are missing a couple of values because a couple nodes are completely unconnected


## We can narrow down the type of plots to display using the "include" argument

plot(bridge_centrality, include="Bridge Strength")

## We can also change how the nodes are ordered in the plot, using the "order" argument

plot(bridge_centrality, include=c("Bridge Strength", "Bridge Betweenness"), order="value")

## You can also plot z-values instead of raw centrality scores:

plot(bridge_centrality, include=c("Bridge Strength", "Bridge Betweenness"), zscore=TRUE)

plot(bridge_centrality, include=c("Bridge Strength", "Bridge Expected Influence (1-step)","Bridge Expected Influence (2-step)"), zscore=TRUE)

## We can extract each type of bridge centrality easily:

bridge_centrality$`Bridge Strength`

bridge_centrality$`Bridge Betweenness`

bridge_centrality$`Bridge Closeness`

bridge_centrality$`Bridge Expected Influence (1-step)`

bridge_centrality$`Bridge Expected Influence (2-step)`

## What about those nice plots where bridges are colored?

## It's a little more tricky, but we can do it

## Select the top 80th percentile Expected Influence:

bridge_strength <- bridge_centrality$`Bridge Expected Influence (1-step)`


## Select the top 80th percentile bridge strength:
```

```
top_bridges <- names(bridge_strength[bridge_strength>quantile(bridge_strength, probs=0.80, na.rm=TRUE)])

## Now create a new community vector where bridges are their own "community"
bridge_num_w1 <- which(names(bridge_strength) %in% top_bridges)
new_communities <- vector()
for(i in 1:length(bridge_strength)) {
  if(i %in% bridge_num_w1) {
    new_communities[i] <- "Bridge"
  } else {new_communities[i] <- community_structure[i]}
}
## And now use that community vector as your "groups" in qgraph!
ne1=qgraph(my_network, layout=L, groups=new_communities)
ne2=qgraph(my_network, layout=L, groups=new_communities)
layout(t(1:2))
plot(ne1)
plot(ne2)
layout(t(1:1))
my_network = n3
community_structure31 <- c(rep("PANSS",2),rep("Neg-Life style",8),rep("ETI", 4))
community_structure=community_structure31
## Now we are ready to look at bridge centrality!
## It's a one-liner with the bridge() function:
require("networktools")
bridge_centrality <- bridge(my_network, communities=community_structure)
bridge_centrality
## It's very easy to get a nice plot of this:
plot(bridge_centrality) ## We are missing a couple of values because a couple nodes are completely unconnected
## We can narrow down the type of plots to display using the "include" argument
plot(bridge_centrality, include="Bridge Strength")
## We can also change how the nodes are ordered in the plot, using the "order" argument
plot(bridge_centrality, include=c("Bridge Strength", "Bridge Betweenness"), order="value")
## You can also plot z-values instead of raw centrality scores:
```

```
plot(bridge_centrality, include=c("Bridge Strength", "Bridge Betweenness"), zscore=TRUE)

plot(bridge_centrality, include=c("Bridge Strength", "Bridge Expected Influence (1-step)","Bridge Expected Influence (2-step)"), zscore=TRUE)

## We can extract each type of bridge centrality easily:

bridge_centrality$`Bridge Strength`

bridge_centrality$`Bridge Betweenness`

bridge_centrality$`Bridge Closeness`

bridge_centrality$`Bridge Expected Influence (1-step)`

bridge_centrality$`Bridge Expected Influence (2-step)`

## Select the top 80th percentile Expected Influence:

bridge_strength <- bridge_centrality$`Bridge Expected Influence (1-step)`

## Select the top 80th percentile bridge strength:

top_bridges <- names(bridge_strength[bridge_strength>quantile(bridge_strength, probs=0.80, na.rm=TRUE)])

## Now create a new community vector where bridges are their own "community"

bridge_num_w1 <- which(names(bridge_strength) %in% top_bridges)

new_communities <- vector()

for(i in 1:length(bridge_strength)) {

  if(i %in% bridge_num_w1) {

    new_communities[i] <- "Bridge"

  } else {new_communities[i] <- community_structure[i]}

}

## And now use that community vector as your "groups" in qgraph!

L4 <- averageLayout(net3)

ne1=qgraph(my_network, layout=L2, groups=new_communities)

layout(t(1:1))

plot(ne1)

#5.MGM, predicability

Data1_norm <- huge.npn(df1)

# Obtain GGM

library(qgraph)

Cor_Study1 <- cor_auto(Data1_norm)

Study1_GLASSO <- qgraph(Cor_Study1, layout = "spring", groups=group.item, graph = "glasso",
```

```
                              tuning = 0.5, sampleSize = nrow(df6), legend.cex = 0.4,
                              color=color,
                              borders=FALSE, theme="colorblind", usePCH=TRUE,title="b)")
WeightMatrix_Study1_GLASSO <- getWmat(Study1_GLASSO)
# Add predictability to the plot
Data1_norm_matrix <- as.matrix(Data1_norm)
p_1 <- ncol(Data1_norm_matrix)
#dim(Data1_norm_matrix)
set.seed(1)
fit_obj1 <- mgm(data=Data1_norm_matrix,
                type = rep('g', p_1),
                level = rep('1', p_1),
                lambdSel = 'EBIC',
                ruleReg = 'OR')

pred_obj1 <- predict(object = fit_obj1,
                     data = Data1_norm,
                     errorCon = 'R2')
a2=pred_obj1$error
library(qgraph)
#print(pred_obj1)
#dev.off()
R2_network1_Spring1 <- qgraph(WeightMatrix_Study1_GLASSO,
                              layout = L,
                              pie = as.numeric(as.character(pred_obj1$error[,2])),
                              pieColor = rep('#377EB8', p_1),
                              labels = colnames(Data1_norm_matrix),
                              groups = group.item, color=color, theme="colorblind",
                              maximum=F, details=F,cut = 0.03,negDashed=F,legend=F, title="a)")
R2_network1_Spring2 <- qgraph(WeightMatrix_Study1_GLASSO,
                              layout = L,
                              pie = as.numeric(as.character(pred_obj1$error[,2])),
```

```
                                        pieColor = rep('#377EB8', p_1),
                                        labels = colnames(Data1_norm_matrix),
                                        groups = group.item, color=color, theme="colorblind",
                                        maximum=F, details=F,cut = 0.03,negDashed=F, title="b)")
#title="a)", groups = community_structure2,cut = 0.03,negDashed=TRUE,layout = L,color=color,legend=F
layout(t(1:2))
plot(R2_network1_Spring1)
plot(R2_network1_Spring2)

Data1_norm <- huge.npn(df3)
# Obtain GGM
library(qgraph)
Cor_Study1 <- cor_auto(Data1_norm)
Study1_GLASSO <- qgraph(Cor_Study1, layout = "spring", groups=group.item2, graph = "glasso",
                                    tuning = 0.5, sampleSize = nrow(df6), legend.cex = 0.4,
                                    color=color,
                                    borders=FALSE, theme="colorblind", usePCH=TRUE,title="b)")

WeightMatrix_Study1_GLASSO <- getWmat(Study1_GLASSO)
# Add predictability to the plot
Data1_norm_matrix <- as.matrix(Data1_norm)
p_1 <- ncol(Data1_norm_matrix)
#dim(Data1_norm_matrix)
set.seed(1)
fit_obj1 <- mgm(data=Data1_norm_matrix,
                    type = rep('g', p_1),
                    level = rep('1', p_1),
                    lambdSel = 'EBIC',
                    ruleReg = 'OR')

pred_obj1 <- predict(object = fit_obj1,
                            data = Data1_norm,
```

```
                                        errorCon = 'R2')
#a2=pred_obj1$error
#a3=pred_obj$error
library(qgraph)
#print(pred_obj1)
#dev.off()
R2_network1_Spring1 <- qgraph(WeightMatrix_Study1_GLASSO,
                                                        layout = "spring",
                                                        pie = as.numeric(as.character(pred_obj1$error[,2])),
                                                        pieColor = rep('#377EB8', p_1),
                                                        labels = colnames(Data1_norm_matrix),
                                                        groups = group.item3, color=color, theme="colorblind",
                                                        maximum=F, details=F,cut = 0.03,negDashed=F,legend=F, title="c)")
data=df3
data <- na.omit(data)
data <- as.matrix(data)
p <- ncol(data)
dim(data)
library(mgm)
set.seed(1)
fit_obj <- mgm(data = data,
                    type = rep('g', p),
                    level = rep(1, p),
                    lambdaSel = 'CV',
                    ruleReg = 'OR',
                    pbar = FALSE)
pred_obj1 <- predict(object = fit_obj,
                                data = data,
                                errorCon = 'R2')
pred_obj2 <- predict(object = fit_obj,
                                data = data,
                                errorCon = 'RMSE')
```

```
a2=pred_obj1$error

a3=pred_obj2$error

Cent=cbind(a2,a3)

#6.Stability of network

b1 <- bootnet(net1, nCores = 8,nBoots = 1000, type = 'nonparametric')

b2 <- bootnet(net1, nCores = 8,nBoots = 1000, type = 'case',

                statistics=c('strength','closeness','betweenness'))

plot(b1, order="sample", plot="area", prop0=F)# confidence intervals

plot(b1, "edge", plot = "difference", onlyNonZero = TRUE, order = "sample") # difference of edges

plot(b1, "strength", plot = "difference") # node strength

plot(b2,statistics=c('strength','closeness','betweenness'))+ theme(legend.title = element_blank())

corStability(b2 )

#7. Bridge EI

nonParametricBoot1 <- bootnet(net1,boots=1000,type="nonparametric", statistics="all", communities=community_structure6)

plot(nonParametricBoot1, statistics="bridgeExpectedInfluence", plot="difference")

plot(nonParametricBoot1, statistics="expectedInfluence", plot="difference")

#Patient

myNetwork <- estimateNetwork(data1,default="cor")

community_structure6 <- c(rep("PANSS",2),rep("BCSS",4), rep("BS", 2),rep("DHQ", 1),rep("PAR", 1),rep("ETI", 4))

MDSnet(qgraph(myNetwork$graph,groups=community_structure6,palette="pastel"))

caseDroppingBoot <- bootnet(myNetwork,boots=1000,type="case", statistics="all", communities=community_structure6)

corStability(caseDroppingBoot)

#Patient

myNetwork <- estimateNetwork(data2,default="cor")

community_structure6 <- list("BCSS" = 1:4,"BS " = 5:6,"DHQ" = 7,"PAR " = 8,"ETI" = 9:12)

MDSnet(qgraph(myNetwork$graph,groups=community_structure6,palette="pastel"))

caseDroppingBoot <- bootnet(myNetwork,boots=1000,type="case", statistics="all", communities=community_structure6)

corStability(caseDroppingBoot)

#Control

myNetwork <- estimateNetwork(data3,default="cor")

community_structure6 <- list("BCSS" = 1:4,"BS " = 5:6,"DHQ" = 7,"PAR " = 8,"ETI" = 9:12)

MDSnet(qgraph(myNetwork$graph,groups=community_structure6,palette="pastel"))
```

```
caseDroppingBoot <- bootnet(myNetwork,boots=1000,type="case", statistics="all", communities=community_structure6)

corStability(caseDroppingBoot)

#Finished
```
